# Reliability, validity and normative data of the timed water swallow test accompanied by sEMG

**DOI:** 10.1007/s00405-024-08884-7

**Published:** 2024-08-06

**Authors:** Dilan Selen Akıl, Serkan Bengisu, Eyüp Sezer, Yakup Krespi, Saime Seyhun Topbaş

**Affiliations:** 1 Department of Speech and Language Therapy, Faculty of Health Sciences, Istanbul Medipol University, Istanbul, Turkey; 2 Department of Speech and Language Therapy, Faculty of Health Sciences, Istanbul Atlas University, Istanbul, Turkey; 3 BAVIM – Stroke Center, Istinye University Liv Hospital, Istanbul, Turkey; 4 Department of Speech and Language Therapy, Faculty of Health Sciences, Fenerbahçe University, Istanbul, Turkey; 5 Department of Neurology Istanbul, Istinye University, Istanbul, Turkey

**Keywords:** Swallowing, Dysphagia, Timed water swallowing test, Electrophysiological evaluation

## Abstract

**Purpose:**

Swallowing disorders are highly interrelated with increased morbidity and mortality rates; therefore, early detection is crucial. Most screening tools rely on subjective observation, thus making objective assessment tools more vital. Timed Water Swallowing Test (TWST) is a screening tool used in the field providing quantitative data. This study aimed to investigate the swallowing parameters in a wide age range by using TWST and to expand the already existing normative data pool accordingly. It is also aimed to examine the reliability of the TWST and assess its validity in stroke survivors.

**Materials and methods:**

This study had a cross-sectional design. TWST carried out simultaneously along with surface EMG and laryngeal sensor on 196 healthy subjects aged 10 to 80 for normative data. Also, TWST carried out 30 patients having a history of recent stroke. Test-retest and inter-rater scoring analysis were used for reliability purposes, while Gugging Swallowing Screen (GUSS) test was used for validity purposes. Additionally, the correlations between the participants’ TWST scores and GUSS scores were examined using the Spearman correlation coefficient.

**Results:**

The normative TWST data of healthy participants are tabulated and presented and their average swallowing capacity was found 13.73 ml/s. Furthermore, the mean swallowing capacity of stroke survivors was found 4.61 ml/s. As a result of validity analyses, a statistically strong and significant relationship was found between GUSS and TWST parameters (*r* = 0.775, *p* < 0.001). Intraclass correlation coefficient (ICC) and correlation values were found between moderate to good agreement between test-retest measurement (ICC = 0.563 to 0.891, *p* < 0.05). Also, the agreement between the raters was found to be significant (ICC = 0.949 to 0.995, *p* < 0.05).

**Conclusion:**

TWST is a valid and reliable screening tool to evaluate dysphagia on given population. Although the test’s performance on healthy individuals is adequate, more research is still needed to confirm that it can be used as a screening tool for stroke.

## Introduction

Dysphagia is a complicated disorder leading persistent coughing, malnutrition and aspiration pneumonia [1]. Early detection of disorder must have a higher priority in order to overcome impending problems [2–4].

In literature, most of the current dysphagia screening tools rely on observation and yield subjective outcomes accordingly [5–7]. It is thought that screening tools used by clinicians must have simple, valid and reliable features [8, 9].

The use of objective quantitative measurement in the evaluation of dysphagia can increase the accuracy of screening and can also be used as a metric indicator of the healing process. For this purpose, Timed Water Swallowing Test (TWST) has been used for objective evaluation of swallowing function [10]. The tool is a modified version of TWST developed by Hughes and Wiles. TWST is a feasible, easy to use functional test of swallowing providing numerical results [11]. It involves a procedure of drinking 100 or 150 ml of water from a cup while patient sits in an upright position. The number of swallows, swallowing-time and swallowing-volumes are recorded during procedure. Both formal and informal data collection methods are utilized during procedure. While observation and laryngeal palpation methods are used as informally; needle or surface electromyography (sEMG) recordings are utilized for more objective results [10, 12].

Volume per swallow (ml; V/S), time per swallow (s; T/S), and swallowing capacity (ml/s; V/T) indices are obtained from this raw data. Swallowing capacity, one of these indices, is thought to yield information about how well a given swallowing is [10].

Normative data were collected from 181 healthy participants in Hughes et al.’s study in 1996, while the same data collected from 480 individuals in Sarve et al.’s study [10, 12]. Following these studies, many studies have been carried out in the field [13–17].

Some studies have suggested that a swallowing capacity below 10 ml/s is a valid threshold to determine swallowing difficulties [12, 13, 18, 19]. While Wu et al. (2004) [14] found that participants with a swallowing capacity below 10 mL/s on the TWST approximately 50% had an impaired swallowing safety, similarly Hägglund et al. (2020) [20] observed that 53% of all stroke participants with swallowing capacity < 10 mL/s had reduced swallowing safety. In the light of these studies, low swallowing capacity from TWST may predict low swallowing function in further assessments [16].

One of the first studies in literature examining the oropharyngeal phase of swallowing electro-physiologically along with sEMG was conducted by Ertekin [21]. Various time-dependent swallowing evaluations were performed in those studies. Included parameters of evaluation were single bolus analysis, dysphagia limit, drinking from a glass, spontaneous breathing at rest and swallowing-breathing relationship during swallowing.

Some other studies carried out included the electro-physiological evaluation of swallowing in patients with Amyotrophic Lateral Sclerosis [22], Wallenberg Syndrome [23] and Spinocerebellar Ataxia [24]. However, those studies lack in sufficient participants and reasonable age range to serve as normative data. Additionally, Şahin et al. [25] examined the swallowing patterns of individuals between the ages of 5–18, and while no age and gender related differences were observed in swallowing time, an increase in the swallowing volume was observed as age increased.

The aim of this study was to examine the performance of the Turkish population on TWST using an EMG device with respect to:

- a) age, gender, and swallowing type parameters in healthy individuals for normative data,
- b) NIHSS score and lesion site parameters in individuals who have had a stroke, and.
- c) determine the validity and reliability of TWST.

## Materials and methods

### Participants

Present study was carried out at stroke unit of Istinye University Liv Hospital between April 2022 and November 2022, and totally 216 individuals participated. Individuals having a history of surgery or thyroid disease, recent sedative, anticholinergic or antidopaminergic drug or alcohol users and pregnant or breast feeders were excluded from the study.

Participants with a range from child to adults were recruited from the general public for this study and stratified into groups based on gender and age. 196 individuals equal in numbers of both gender between the ages of 10–80 were included for the healthy group of the study (Table 1). They were included using WHO disability checklist [26], the EAT-10 [27] screening tool and having an ability that sitting upright independently. However, individuals with a problem that would affect swallowing function, recent use of sedative, anticholinergic or antidopaminergic drugs or alcohol, and those who were pregnant or breastfeeding were not included in the study.

Due to after the Covid-19 period and measures, the number and age range of our participants are limited. The healthy group was included in the study to provide normative data and test-retest and inter-rater reliability about all age groups.

**Table 1:** Healthy participants’ numbers according to age and gender

Age Range	Women	Men
10.0-11.11	14	14
12.0-14.11	14	14
15.0-17.11	14	14
18.0-24.11	14	14
25.0-39.11	14	14
40.0-59.11	14	14
60.0–80.0	14	14

The patient group was consisted of individuals with a history of various types of strokes, hospitalized in the acute stroke unit and were not received swallowing treatment. A total of 30 individuals both equal in numbers and genders were included in this group. Patients’ ages were ranged from 60 to 80. All evaluations were performed during the screening before advanced evaluation. Obtained data was used to determine the criterion validity (See Table 2).

**Table 2:** Number of patients with stroke

**Chronological age average**	
Women	70,63
Men	70,05
**Number of participant**	
Women	15
Men	15
**Lesion site**	
Left	16
Right	14
**NIHSS Average**	
Women	4,8
Men	3,93

### Design

The study has a cross-sectional design. Quantitative analyzes of each swallowing indices were performed for each participant and average data for the healthy and patient groups were presented accordingly as well.

### Materials

Demographic information from, WHO 10-Point Disability Checklist [26] and Eating Assessment Tool (EAT-10) [27, 28] tools were used to include participants in this study. In the study, Gugging Swallowing Screen (GUSS) [5], a screening tool with proven reliability used to determine the severity of dysphagia and the risk of aspiration in acute stroke patients, was used for reliability analyses.

#### WHO 10-Point disability checklist

The Checklist developed by WHO previously was modified by Singhi et al. [26]. The checklist includes 10 components that addresses linguistic, cognitive, sensory, behavioral, and other issues of the participants.

#### EAT-10

EAT-10 is one of the most frequently used methods for dysphagia screening and consists of 10 questions developed by Belafsky et al. [28]. The questions are scored between 0 and 4 (0 = no problem, 4 = severe problem) and total score of 3 and above means a risk for dysphagia. The scale is a self-administered and symptom-specific tool and it measures the severity of swallowing disorder, quality of life and treatment effectiveness.

#### GUSS

Test is used to determine the severity of dysphagia and aspiration risk in acute stroke patients with proven reliability [5]. Practitioners use first semi-solid, then liquid and solid boluses respectively and patients should swallow them. On the GUSS, the patients are scored over 20 points and they need to take maximum score of 20 so as to considered typical swallowing [29]. The GUSS was administered in the same way to participants with a history of stroke to determine the validity of the TWST. Then, compatibility of the TWST and GUSS tests was analyzed [5, 10].

#### NIHSS Scale

The National Institutes Health Stroke Scale (NIHSS) is a scale used in emergency and neurology services for stroke patients, which examines neurologic functions and gives an idea about prognosis. The NIHSS can be applied in a very short time and the total score is between 0 and 42 [30]. In general, a linear relationship is expected between NIHSS score and patient clinic [31]. In our study, it was applied to 30 stroke survivors in need of further evaluation.

#### TWST

TWST is a test of water swallowing. In this test, swallowing time, swallowing volume and number of swallows data are required to calculate V/S, T/S and V/T. Although it is not necessary to use an additional equipment in TWST, using a device like EMG has an advantage in terms of ensuring objectivity. The screening tool does not have specific test items and so as to obtain the 3 indices of swallowing, stopwatch (swallowing time), volume meter (swallowing volume), video camera and submental EMG (SM-EMG) and/or laryngeal/nasal sensor (number of swallows) are used [10].

TWST was performed twice using 100 ml of International Dysphagia Diet Standardization Initiative (IDSSI) level-0 consistency for each participant [32].

### Surface EMG

**Fig. 1:**
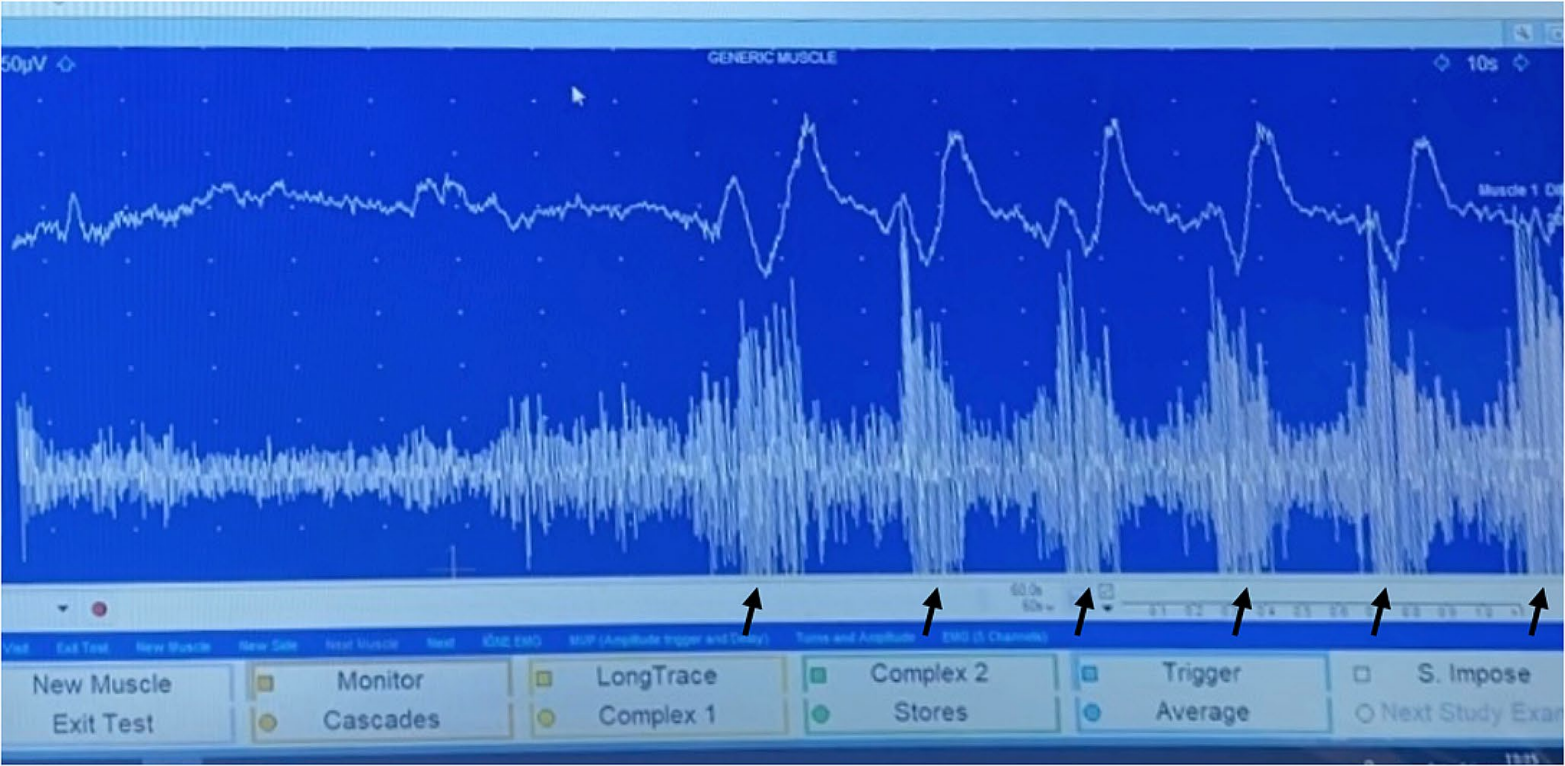
The recording images on the oscilloscope

It is a non-invasive procedure detecting, recording and interpreting of the electrical activity of the muscle groups which is placed upon [33]. The Medeley Synergy SINC5-C EMG device, sEMG electrodes and a laryngeal sensor were used to implement TWST. In this study, a two-channel EMG device was used and the number of swallows obtained from each channel confirmed each other (Fig. 1). In the first channel of EMG, there was a laryngeal sensor plugged in to detect the vertical movements of the thyroid cartilage and in the second channel of EMG, SM-EMG cable was plugged in to measure the activation of suprahyoid muscle complex [34]. The black arrows in the Fig. 1 show each swallowing action. The moment of intersection in the first and second channel of the EMG indicates the swallowing action.

SM-EMG tracing is a way of examination submental muscle activity at the onset of swallowing. SM muscle complex, namely mylohyoid, geniohyoid and anterior digastric muscles activation triggered by each swallow. In addition, a laryngeal sensor placed on the coniotomy area allows us to visualize each pharyngeal swallowing movement in the first channel of EMG during swallowing [34].

While one can trace the laryngeal movement activity via the first channel of EMG, SM muscle complex activation for the same patient may be traced simultaneously via the second channel of EMG. By this way, it’s possible to get two measurements of each swallowing for each individual allowing us to calculate the exact number of swallowing with higher accuracy. In this study, sEMG was used to obtain more reliable data. Surface EMG is not used in the normal TWST procedure.

### Laryngeal sensor

Laryngeal sensor which was developed by Pehlivan [34] in 1996 is used to determine the onset and ending of the pharyngeal swallowing movement. The sensor is used along with EMG device and not only detects laryngeal movements, but also detects the swallowing sounds produced during the transfer of the bolus and helps to calculate the number of swallows with SM-EMG [34].

### Procedure

Two speech and language therapist (SLT), one of whom experienced in swallowing, were involved in the present study. The whole procedure was carried out together. First of all, the purpose of the study was explained to the participants and then detailed information about the interventions was given. Individuals who agreed to participate signed a consent form and were included in the study.

**Fig. 2:**
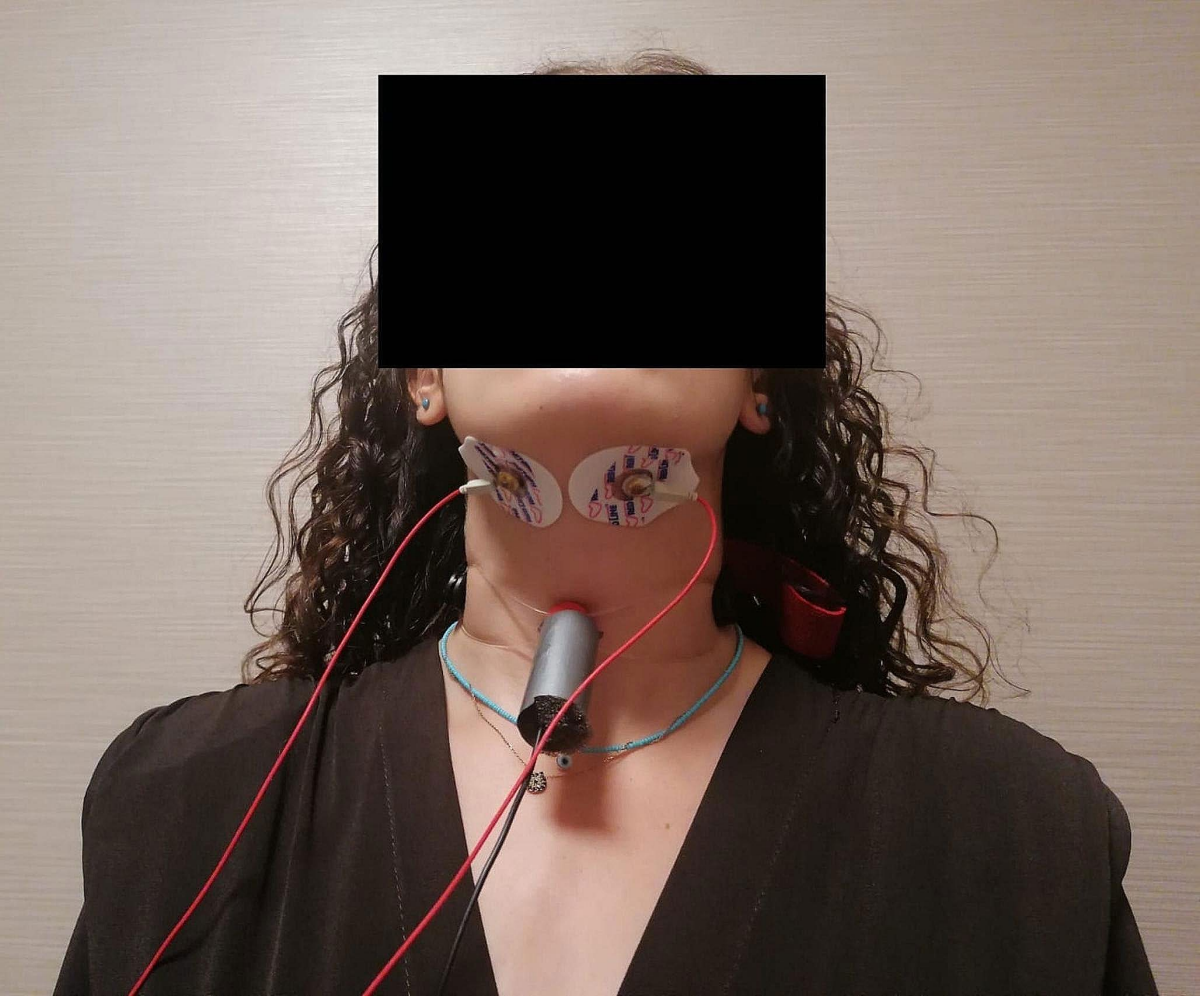
Example of laryngeal sensor and electrode placement

Before the assessment, 2 glasses of water (100 ml each) at room temperature, a camera for lateral video recording, a stopwatch, 2 pediatric electrocardiography (ECG) electrodes, laryngeal sensor and EMG device were prepared. Participants were seated on a chair and target areas were marked for laryngeal sensor and electrode placements. The laryngeal sensor was placed on the ‘coniotomy’ area between the thyroid and cricoid cartilage and fixed to the neck with a rubber band during the application [35]. SM-EMG electrodes (pediatric ECG) were also placed under the chin, 2 cm distance from the midline to the right and left. In SM-EMG, surface electrodes are placed for live recording, while the third is attached to the neck as the ground electrode and still another one was connected to the wrist to reduce the signal/noise ratio. The placement of the laryngeal sensor and EMG electrodes on the participants is shown as an example in Fig. 2.

Then, the signals were filtered between 0.2 Hz and 30 Hz in the first channel, and filtered between 100 Hz and 10 kHz in the second channel accordingly in EMG device. Simultaneous recording was made available from the two channels of EMG using the delay line technique while drinking water. The sweep time was chosen as 50 s, and the records were windowed in 10 seconds using the ‘long trace’ technique [35].

All data were collected simultaneously along with laterally placed video-recorder during the evaluation in a silent room. Participants were given 2 glasses of room-temperature water each containing 100 ml accordingly. They were asked to drink a total of 200 ml of water, first “normally” and afterword “as fast as you can till you consume all” on command.

Swallowing time was decided on using a stopwatch starting with the moment of the glass touched the lips till a sustained /a/ phonation was produced after final swallow. SM-EMG and laryngeal sensor data channels were simultaneously used via EMG device to decide each number of swallowing along with video-recording [10, 12]. Finally, the volume of swallow was prepared beforehand by us (100 ml).

Using the data of our present study (the number of swallows, the time of swallow and the volume of swallow) three swallowing indices were calculated; V/S, T/S and V/T. V/S was calculated by dividing the total volume consumed (100 ml) by the total number of swallows while T/S was calculated by dividing the total swallowing time by the number of swallows. Finally, swallowing capacity (V/T) was calculated by dividing the V/S by T/S [10, 12].

### Reliability

#### Test-retest reliability

The test-retest method is the application of an assessment scale twice to the same participants, in the same situation and within a certain period of time. The values calculated as a result of the two applications are analyzed to find the correlation coefficient that shows the agreement between the applications [36].

In our study, TWST was applied accompanied by EMG to 30 participants selected by the researcher SLT at two different times for test-retest reliability. Participants’ retesting times range from an hour to one day intervals.

#### Inter-rater reliability

The inter-rater reliability method is used to express the correlation between multiple, independent observers evaluating cases in the same sample [37].

Evaluations were performed independently by the two observers. In addition, the two evaluators were blind to each other and had no information about the patients. In our study, the principal investigator and another qualified SLT evaluated 30 participants simultaneously in the same place. During this evaluation, TWST was applied accompanied by EMG. Raters simultaneously recorded each patient’s number of swallows, duration of swallow and the volume swallowed at each trial and converted these data into V/S, T/S and V/T indices accordingly.

### Validity

Validity means that the measurement tool used is appropriate for the characteristic to be measured, the data are useful for the purpose and fully reflect the nature of the characteristic to be measured [37]. Criterion validity was used in this study. Criterion validity is the comparison of the results obtained with the existing scale with another scale determined as a standard [5, 10].

In our study, concurrent validity analysis was used for criterion validity. GUSS was used as the criterion scale. The GUSS was used consecutively with the TWST to assess concurrent validity. Both screening tools were applied to 30 patients hospitalized in the stroke unit. Patients were selected by investigator according to inclusion criteria. While the TWST [10] application was carried out by the responsible SLT, the GUSS [5] screening tool was carried out by another experienced SLT. In practice, being in post-24-hour after stroke, able to sit in a wheelchair and following commands was taken into consideration. Water was used for liquids, yoghurt for semi-solids and bread for solid consistency assessments.

### Statistical analyses

The statistical analyses were conducted using SPSS software version 26.0 (IBM SPSS Inc., Chicago, IL). T-test was used to compare data between two independent groups and one-way analysis of variance (ANOVA) test was used to compare data among more than two independent groups. Scheffe test was used as a complementary post-hoc analysis. Pearson correlation was applied for the continuous variables of the study.

The test-retest reliability of this study, independent screenings was assessed using the intraclass correlation coefficient (ICC) and 95% confidence intervals. Also, for inter-clinician reliability, a two-way random effect model was selected and the ICC was calculated with 95% confidence intervals. For ICC, positive value results ranging from 0 to 0.5 indicate poor agreement; 0.5 to 0.75, moderate agreement; 0.75 to 0.9, good agreement; and 0.9 to 1, excellent agreement [38, 39]. Additionally, the correlations between the participants’ TWST scores and GUSS scores were examined using the Spearman correlation coefficient.

## Results

196 healthy participants were included in the study for description of normative data, test-retest and inter-rater reliability examination. Moreover, 30 stroke survivors were included in the study for criterion validity.

### Healthy participants

First of all, normative TWST data were collected for normal and rapid swallowing tasks in the healthy group, including women, men and the whole group. Descriptive statistics (min, max, mean [± standard deviation]) were calculated for V/S, T/S and V/T in each group, regardless of age group (Table 3).

For the normal swallowing task, the lowest V/S was 5.55 ml, the lowest T/S was 0.68 s and the lowest V/T was 3.88 ml/s. For the fast-swallowing task, the minimum V/S was 6.66 ml, the minimum T/S was 0.71 s and the minimum V/T was 5.44 ml/s.

Also, for the normal swallowing task, the highest V/S is 50 ml, the highest time per swallow is 3.23 s and the highest V/T is 33.3 ml/s. For the fast-swallowing task, the maximum V/S is 100 ml, the maximum T/S is 3.45 s and the maximum V/T is 35.71 ml/s.

**Table 3:** TWST data obtained from healthy participants

		Normal Swallow				Fast Swallow			
		Min.	Max.	Mean	SD	Min.	Max.	Mean	SD
V/S (ml)	Women	5.88	25	15.43	4.75	6.66	50	20.47	7.35
	Men	5.55	50	19.54	7.65	8.33	100	27.05	11.98
	Both	5.55	50	17.48	6.68	6.66	100	23.76	10.45
T/S (s)	Women	0.71	3.23	1.32	0.37	0.76	2.7	1.23	0.38
	Men	0.68	2.76	1.31	0.33	0.71	3.45	1.26	0.38
	Both	0.68	3.23	1.31	0.35	0.71	2.7	1.24	0.38
V/T (ml/s)	Women	3.88	24.03	12.21	4.12	5.44	32.05	16.98	5.05
	Men	4.52	33.33	15.26	5.74	7	35.71	21.32	5.88
	Both	3.88	33.33	13.73	5.21	5.44	35.71	19.15	5.88

Min: Minimum, Max: Maximum, SD: Standard Deviation

### Effects of age

One-way ANOVA was used to establish the correlation between ages and TWST measurements. In the study, no significant difference was observed in V/S and V/T data according to ages (*p* > 0.05). A significant difference was observed in the participants’ T/S measurements according to ages only in the normal swallowing task (*p* < 0.01) (Table 4).

In the normal swallowing trial, the highest T/S values were in the 25-39.11 age group. T/S measurements obtained from this group are significantly different from all younger age group’s measurements. Additionally, it was observed that starting from the 25-39.11 age group T/S data tend to decrease as age increased.

**Table 4:** V/S, T/S and V/T data distribution on the age parameter during normal and fast swallowing trials

Trials	Age Groups	n	V/S (ml)			T/S (s)			V/T (ml/s)		
			Mean	SD	p	Mean	SD	p	Mean	SD	p
Normal Swallow	10.0-11.11	28	15.697	7.259	0.144	1.197	0.401	0.000**	13.727	6.176	0.706
	12.0-14.11	28	16.713	4.338		1.128	0.308		15.321	4.216	
	15.0-17.11	28	17.002	5.838		1.262	0.291		13.479	3.550	
	18.0-24.11	28	16.019	5.455		1.283	0.235		12.873	5.416	
	25.0-39.11	28	20.186	9.449		1.475	0.441		14.035	6.313	
	40.0-59.11	28	18.248	6.258		1.449	0.294		13.207	5.707	
	60.0–80	28	18.559	6.567		1.423	0.354		13.557	4.756	
Fast Swallow	10.0-11.11	28	18.829	8.819	0.069	1.141	0.298	0.218	16.533	5.565	0.072
	12.0-14.11	28	22.491	8.788		1.301	0.552		17.812	5.065	
	15.0-17.11	28	24.005	9.720		1.192	0.376		20.224	5.224	
	18.0-24.11	28	23.737	8.237		1.172	0.251		20.481	6.428	
	25.0-39.11	28	27.776	17.355		1.286	0.427		20.743	6.074	
	40.0-59.11	28	23.963	7.391		1.290	0.262		18.948	6.008	
	60.0–80	28	25.540	8.337		1.381	0.420		19.346	6.091	

SD: Standard Deviation, *p < 0.05, **p < 0.01 is considered significant

### Effects of gender

One-way ANOVA was used to determine the correlation between gender and TWST measurements. Analysis of swallowing indices according to gender parameter are given in Table 5. There was a significant difference in the participants’ V/S and V/T measurements on gender parameter (p < 0.01). On both normal swallowing trials men’ V/S and V/T measurements were higher than women’ V/S and V/T measurements.

**Table 5:** V/P, T/P and V/T data distribution on the gender parameter during normal and fast swallowing trials

Trials	Groups	n	V/S (ml)			T/S (s)			V/T (ml/s)		
			Mean	SD	p	Mean	SD	p	Mean	SD	p
Normal Swallow	Women	98	15.436	4.757	0.000**	1.322	0.374	0.83	12.217	4.127	0.000**
	Men	98	19.542	7.660		1.311	0.339		15.269	5.747	
Fast Swallow	Women	98	20.471	7.358	0.000**	1.237	0.387	0.582	16.989	5.051	0.000**
	Men	98	27.055	11.990		1.267	0.384		21.321	5.883	

SD: Standard Deviation, *p < 0.05, **p < 0.01 is considered significant

However, participant’s T/S measurements on normal swallowing measurements (*p* = 0.83) and fast swallowing measurements (*p* = 0.582) did not differ significantly according to gender parameter (*p* > 0.05).

### The effect of age-gender interaction on measurements

In our study, the effects of age and gender independent variables on V/S, T/S and V/T in normal and rapid swallowing tasks were analyzed by Two-Way Analysis of Variance. In the results obtained, there is an effect of age-sex interaction only for the measurement of swallowing capacity in the normal swallowing task (*p* < 0.05).

### Effects of swallowing type

Dependent Groups T Test was used to investigate the difference between swallowing types. While TWST’s all three components showed significant differences on both normal and fast swallowing trials (*p* < 0.05), V/S and V/T data showed highly significant differences as well in the study (*p* < 0.01) (Table 6).

**Table 6:** Effect of age-gender interaction on swallowing measurements

Trials	*n*	Normal Swallow		Fast Swallow		*p*
		Mean	SD	Mean	SD	
V/S (ml)	196	17.489	6.684	23.763	10.457	0.000**
T/S (s)	196	1.317	0.356	1.252	0.385	0.035*
V/T (ml/s)	196	13.743	5.220	19.155	5.884	0.000**

SD: Standard Deviation, *p < 0.05, **p < 0.01 is considered significant

### Participants having stroke

Table 7 presents TWST data on 30 people aged 60–80 years who had had a stroke in both male and female gender groups. Only normal swallowing data were collected from this group because of swallowing security.

**Table 7:** TWST data obtained from participants having stroke

Trials		Min.	Max.	Mean	SD
V/S (ml)	Women	1.56	25	9.78	6.3
	Men	4.54	50	12.64	11.61
	Both	1.56	50	11.21	9.29
T/S (s)	Women	1.46	7	2.6	1.37
	Men	1.1	5.63	2.69	1.21
	Both	1.1	7	2.64	1.27
V/T (ml/s)	Women	0.59	11.36	4.21	2.77
	Men	0.88	12.62	5.01	3.1
	Both	0.59	12.62	4.61	2.88

Min: Minimum, Max: Maximum, SD: Standard Deviation

### Effects of NIHSS score

One-way ANOVA was used to determine whether there was a significant difference between the NIHSS score obtained from the participants and swallowing capacity. The results of the analysis are given in Table 8. As a result, the V/S, T/S and V/T data of the participants in the normal swallowing task did not differ significantly according to the NIHSS score (*p* > 0.05).

**Table 8:** The relationship between TWST data and NIHSS score in stroke survivors

Trials	NIHSS Score	
V/S (ml)	r	-0.226
	p	0.230
T/S (s)	r	-0.144
	p	0.448
V/T (ml/s)	r	-0.140
	p	0.459

Pearson Correlation Analysis, r: Correlation Coefficient, *p < 0.05, **p < 0.01 is considered significant

### Effects of lesion site

One-way ANOVA was used to determine whether there was a significant difference between the affected side and V/T (Table 9). In conclusion, the V/S, T/S and V/T measurements of the participants in the normal swallowing task did not show a significant difference compared to the affected side (*p* > 0.05).

**Table 9:** The relationship between TWST data and lesion sites in stroke survivors

Trials	Lesion Site	*n*	Mean	SD	*p*
V/S (ml)	Right	14	10.754	11.841	0.806
	Left	16	11.612	6.721	
T/S (s)	Right	14	2.649	1.447	0.997
	Left	16	2.647	1.148	
V/T (ml/s)	Right	14	4.322	3.236	0.615
	Left	16	4.873	2.694	

Independent Groups T-Test, SD: Standard Deviation, *p < 0.05, **p < 0.01 is considered significant

### Reliability

ICCs was used to test in-session test-retest reliability (Table 10). Measurements made with ICC, ICC for each swallowing index measurement ranged between 0.563 and 0.891, and in-session test-retest reliability is considered to be good to excellent.

**Table 10:** Reliability on test-retest trials

Trials		n	Test	Re-test	ICC (95% CI)	p
			Mean ± SD	Mean ± SD		
Normal Swallow	V/S (ml)	30	18.99 ± 7.13	18.21 ± 6	0.854 (0.693–0.931)	0.0001**
	T/S (s)	30	1.34 ± 0.49	1.23 ± 0.29	0.687 (0.343–0.851)	0.001**
	V/T (ml/s)	30	14.57 ± 4.9	14.93 ± 4.28	0.891 (0.771–0.948)	0.0001**
Fast Swallow	V/S (ml)	30	22.45 ± 9.25	22.12 ± 8.72	0.808 (0.597–0.909)	0.0001**
	T/S (s)	30	1.22 ± 0.33	1.37 ± 0.62	0.563 (0.082–0.792)	0.015*
	V/T (ml/s)	30	18.21 ± 5.31	16.51 ± 4.09	0.821 (0.621–0.914)	0.0001**

SD: Standard Deviation, ICC: Intraclass Correlation Coefficient, CI: Confidence Interval, *p < 0.05, **p < 0.01 is considered significant

Correlation analysis was used to test inter-rater reliability and the results are given in Table 11. In ICC measurements, inter-rater reliability was thought to be good to excellent, varying between 0.949 and 0.995.

**Table 11:** Inter-rater reliability

Trials		n	1st Rater	2nd Rater	ICC (95% CI)	p
			Mean ± SD	Mean ± SD		
Normal Swallow	V/S (ml)	30	16.33 ± 4.46	16.48 ± 4.46	0.995 (0.989–0.997)	0.0001**
	T/S (s)	30	1.35 ± 0.25	1.34 ± 0.27	0.971 (0.939–0.986)	0.0001**
	V/T (ml/s)	30	12.28 ± 3.52	12.52 ± 3.67	0.989 (0.977–0.995)	0.0001**
Fast Swallow	V/S (ml)	30	22.49 ± 8.61	22.82 ± 8.61	0.992 (0.983–0.996)	0.0001**
	T/S (s)	30	1.23 ± 0.27	1.2 ± 0.24	0.949 (0.892–0.976)	0.0001**
	V/T (ml/s)	30	18.16 ± 5.06	18.85 ± 5.26	0.977 (0.952–0.989)	0.0001**

SD: Standard Deviation, ICC: Intraclass Correlation Coefficient, CI: Confidence Interval, *p < 0.05, **p < 0.01 is considered significant

### Validity

Convergent validity agreement between GUSS and TWST tests was examined using Spearman Correlation Coefficient (Table 12). A statistically significant and very strong positive relationship was found between GUSS and V/T scores in TWST (*r* = 0.775, *p* < 0.0001).

**Table 12:** Data for validity on patients having stroke

Group	GUSS Score	V/T Score	*r*	*p*
	Mean ± SD	Mean ± SD		
Participants having stroke	10.4 ± 2.41	4.61 ± 2.91	*r* = 0.775	0.0001**

Spearman Correlation Coefficient, SD: Standard Deviation, r: Correlation Coefficient, *p < 0.05, **p < 0.01 is considered significant

## Discussion

The current study revealed the effects of age, gender and swallowing type parameters on swallowing indices and also included validity and reliability. It is thought that the data obtained may be used as an indicator of the need for further evaluation of individuals in terms of swallowing disorders and to decide on their therapy progress.

### Healthy participants

#### Effects of age

In this study, although V/S measurements did not show a significant difference according to age parameter, V/S values increased up to the age of 40, but showed a tendency to decrease after the age of 40. The reason for this is that the cavity volumes of the larynx and hypopharynx decrease, smaller spaces are observed in the pyriform sinuses [40], oral transmit time increases and it takes longer to reach maximal tongue pressure with aging [41–43]. It is thought that there may be a slow triggering of the swallowing reflex [44] and an increase in the space between the hyoid bone and the mandible with age [16]. This finding is similar to the data of Sarve et al., which reported in the literature that V/S values increases with age, but begin to decrease after the age of 60 [10].

In the present study, a significant difference was observed in T/S values according to age, and the data of 25.0-39.11 age groups’ is higher than all groups. The reason for this observed effect is may be; due to the full maturation of laryngeal and pharyngeal structures and enabling them to reach the maximum transit times available during this period [45]. Additionally, our findings are consistent with a study in the literature by Inamoto et al. [45] that gender, height and age parameters each have an effect on swallowing [40]. However, no significant difference was observed in terms of T/S between the groups under the age of 25. It is thought that the reason for this finding may be that maturation process of the laryngeal and pharyngeal structures involves individual differences [45].

Sarve et al. revealed that swallowing time decreases with age, but tend to increase after the age of 60 [10]. In contrast with this, Hughes and Wiles, Sella-Weiss and Alfonsi et al., observed that the average swallowing time increased with aging both in men and women [12, 15, 44].

Although there was no significant difference in V/T data by age, a decrease was observed after the age of 40. The reason for this finding is thought to be the use of relative results instead of absolute ones. Similarly, Sella-Weiss also states in his study that using relative measurements such as V/S and T/S could lead to such results [15]. Finding of age-related V/T decrease in our study is similar to other studies in the literature [10, 12, 16].

#### Effects of gender

Significant differences were observed in V/S and V/T measurements according to gender parameter. In both swallowing tasks, higher values were obtained from men’s measurements. It is thought that current results may be due to the fact that men’s vocal tracts are longer than women’s and oropharyngeal cavity volume in men higher than in women [46]. This finding in our study is similar to the other studies in the literature [10, 12, 15, 17].

No significant difference was observed in T/S measurements according to gender parameter. Similarly, in some studies in the literature, no significant difference was observed in T/S values [10, 12, 15]. However, Hiss et al. [47] reported that women have a longer duration of swallowing apnea than men suggesting that as the bolus volume increases, the duration of swallowing apnea increases as well.

#### Effects of swallowing type

Significant differences were observed in V/S, T/S and V/T measurements according to swallowing type parameters. While V/S and V/T values were higher in fast swallowing than in normal swallowing, T/S values were found to be lower in fast swallowing. Sarve et al. [10] also noted significant differences between normal and fast swallowing tasks for V/S, and a higher V/S value was observed in fast swallowing.

### Participants having stroke

#### Effects of NIHSS score

In our study, according to the NIHSS scores of the participants; V/S, T/S and V/T measurements did not show significant differences. The reason for this finding is thought to be due to the limited number of participants.

In the study of Okubo et al. [48] on the prediction of swallowing disorder by the NIHSS score, it was found that the NIHSS was highly sensitive (88%) and specific (85%) in detecting dysphagia, and 12 points was determined as the cut-off value for dysphagia in the NIHSS score [48]. Similarly, Henke et al. [49] compared the NIHSS score with clinical swallowing assessment to determine early and easily assessable determinants of dysphagia in patients with acute ischemic stroke in their 2017 study. In the results, it was reported that stroke severity in terms of NIHSS is a simple and reliable indicator of dysphagia and patients with NIHSS value ≥ 5 should be referred to a professional swallowing examination rapidly [49]. In the study conducted by Kassem et al. [50] in 2022, an NIHSS cut-off value of 4.5 with a sensitivity of 0.77 and specificity of 0.77 for optimal discrimination between patients with and without dysphagia emerged. In addition, an NIHSS cut-off value of 5.5 was obtained to differentiate patients with and without pneumonia [50]. In our study, no correlation was observed between NIHSS score and swallowing capacity, which is consistent with the literature.

#### Effects of lesion site

In our study, V/P, T/S and V/T measurements of the participants did not differ significantly according to the affected side.

In the literature, there are studies supporting that pharyngeal phase is more affected in right hemisphere damage and oral phase is more affected in left hemisphere damage [51, 52], but left-brain stroke increases the risk of pneumonia compared to the right side [53]. In addition, it has been reported that the prognosis is worse in patients with bilateral lesions [51, 52].

In the study conducted by Şapçıoğlu [54] in 2019, no difference was found between the affected side and swallowing measurements of participants with right and left hemisphere-affected hemiplegia in terms of 100 ml water swallowing performance and dysphagia severity. Our study is consistent with this reported finding.

In addition, as a result of the same study conducted by Şapçıoğlu [54], it was found that patients whose right hemisphere was affected had increased sequential water swallowing ability, increased swallowing volume and decreased swallowing rate when dysphagia severity decreased. In patients whose left hemisphere was affected, it was found that when dysphagia severity decreased, sequential water swallowing ability increased. It was found that variables related to water swallowing performance (especially those related to volume) were directly related to dysphagia severity, regardless of the affected hemisphere in right or left hemisphere involvement. Moreover, swallowing volume and speed are more affected by dysphagia severity in individuals with right hemisphere involvement. As a result of the study, it was suggested that rehabilitation programs should be designed taking into account hemisphere involvement.

### Reliability

In this study, ICC was calculated to determine the agreement between test-retest measurements. As a result of the analysis, it was determined that the test-retest measurements (ICC) values varied between 0.563 and 0.891 (*p* < 0.05). It was concluded form the data that, the scale produces reliable measurements over a short period of time.

Previous similar studies conducted on neurologically affected patient groups have reported high test-retest correlation values [13, 14, 19]. In the literature, Sarve et al. test-retest reliability was found to be very high, with Cronbach α values between 0.94 and 0.99. ICC values range between 0.83 and 0.98, indicating a high level of internal consistency [10]. Hägglund et al. [16] also showed that TWST performance obtained from healthy elderly individuals had a high inter and intra-rater agreement when rated asynchronously form the video recordings. In Sella-Weiss’ [15] study, ICC values for within-session test-retest reliability ranged between 0.822 and 0.904 indicating that test-retest reliability is good to excellent within-session.

Correlation analysis was applied to determine inter-rater reliability in our study. The compatibility between the raters was found to be significantly high, and intra-class correlation values between the raters were also found to be high (ICC = 0.949 to 0.995, *p* < 0.05). It has been shown in the literature that TWST has a high inter-rater reliability [12–14, 19].

Sarve et al.’s [10] study, the agreement between raters was calculated and Cronbach α value was found to be > 0.9. This indicates a high level of internal consistency. ICC values for all measurements were calculated as 0.964, indicating an excellent internal reliability.

### Validity

In this study, The GUSS test, a standard screening tool, was used to determine the convergent validity of the TWST. A statistically significant and very strong positive relationship was found between GUSS and TWST scores. It is observed that as the participants’ GUSS scores decreases, their TWST scores tend to decrease as well.

The results are similar to the studies of Hughes et al. [12]. Researchers have associated reduction in V/T and mean V/S values with a number of findings that may be an indicator of swallowing problems, providing further evidence of the measurement validity of these indices in patients with a motor neuron disease. Sarve et al. which concluded that the ICC value between instrumental and behavioral measurements of the number of swallows was 0.85 with a 95% interval similarly [10].

### Limitations

The first limitation of this study is that the number of participants was limited. However, this happened because of Covid-19 and its effects on people. Also, the amount of water (100 ml) given to patients in TWST was limited. For future studies, it is recommended to include larger participants and examine different disease groups separately.

## Conclusion

The current study revealed normative values for TWST on V/S, T/S and V/T parameters in Turkish population. The average V/T data obtained from healthy individuals between the ages of 10.0 and 80 was 13.73 ml/s in the normal swallowing trial and 19.15 ml/s in the fast-swallowing trial.

Moreover, in addition to being a valuable screening tool for dysphagia, it is thought that TWST can be also used as a quantitative indicator of the progress made in therapy.

In the light of the data obtained, the data pool for TWST was expanded, thus proving the accuracy of the data in the literature once again.

## Data Availability

The data used to support the findings of this study are included within the article.
